# 0.5 V Versatile Voltage- and Transconductance-Mode Analog Filter Using Differential Difference Transconductance Amplifier

**DOI:** 10.3390/s23020688

**Published:** 2023-01-06

**Authors:** Tomasz Kulej, Montree Kumngern, Fabian Khateb, Daniel Arbet

**Affiliations:** 1 Department of Electrical Engineering, Czestochowa University of Technology, 42-201 Czestochowa, Poland; 2 Department of Telecommunications Engineering, School of Engineering, King Mongkut’s Institute of Technology Ladkrabang, Bangkok 10520, Thailand; 3 Department of Microelectronics, Brno University of Technology, Technická 10, 601 90 Brno, Czech Republic; 4 Faculty of Biomedical Engineering, Czech Technical University in Prague, Nám. Sítná 3105, 272 01 Kladno, Czech Republic; 5 Department of Electrical Engineering, University of Defence, Kounicova 65, 662 10 Brno, Czech Republic; 6 Faculty of Electrical Engineering and Information Technology, Slovak University of Technology, 81219 Bratislava, Slovakia

**Keywords:** differential different transconductance amplifiers (DDTA), analog filter, oscillator, analog circuit

## Abstract

In this work, a new versatile voltage- and transconductance-mode analog filter is proposed. The filter, without requiring resistors, employs three differential-difference transconductance amplifiers (DDTAs) and two grounded capacitors, which is suitable for integrated circuit implementation. Unlike previous works, the proposed filter topology provides: (1) high-input and low-output impedances for a voltage-mode (VM) analog filter, that is desirable in a cascade method of realizing higher order filters, and (2) high-input and high-output impedances for a transconductance-mode (TM) analog filter without any circuit modification. Moreover, a quadrature oscillator is obtained by simply adding a feedback connection. Both VM and TM filters provide five standard filtering responses such as low-pass, high-pass, band-pass, band-stop and all-pass responses into single topology. The natural frequency and the condition of oscillation can be electronically controlled. The circuit operates with 0.5 V supply voltage. It was designed and simulated in the Cadence program using 0.18 µm CMOS technology from TSMC.

## 1. Introduction

**Figure 1:**
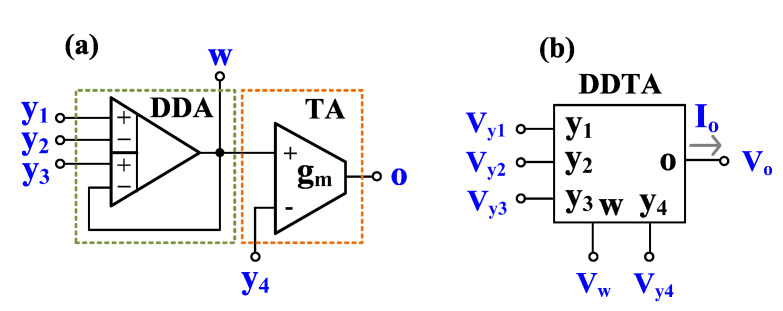
DDTA, (**a**) possible realization of DDTA, (**b**) electrical symbol.

The differential difference amplifiers (DDA) are versatile analog building blocks that offer two differential input ports, high-input impedance, and low-output impedance [1,2,3]. If an ideal DDA (infinite open-loop gain) operates in negative feedback configuration, it can work as summing and subtracting amplifier with unity gain. This ability has been used to increase the performance of new devices such as a differential difference current conveyor (DDCC) [4], differential difference current conveyor transconductance amplifier (DDCCTA) [5], or differential difference transconductance amplifier (DDTA) [6]. The properties of DDCC are similar to those of the conventional second-generation current conveyor (CCII) [7] except the input port that offers arithmetic operation. Thus, DDCC-based filters require resistors and its natural frequency cannot be tuned electronically, for example, see [8,9,10,11,12]. To obtain electronic tuning ability, the DDTA has been presented. The DDTA is a cascade connection of DDA and a transconductance amplifier (TA), as shown in Figure 1a, and its electrical symbol is shown in Figure 1b. Compared with DDCCTA, DDTA is more compact. The DDTA provides addition and subtraction of the voltages V_y1_, V_y2_, V_y3_ at the w-terminal, namely, V_w_ = V_y1_ − V_y2_ + V_y3_. This terminal usually provides low-impedance level (results from negative feedback of DDA), which is suitable for output terminals of voltage-mode circuits. The o-terminal provides output current which is the product of the differential voltage V_w_-V_y4_ and the transconductance g_m_, i.e., I_o_ = g_m_(V_w_-V_y4_). Thus, an electronic tuning ability of DDTA can be obtained by tuning $g_{m}$. The ideal characteristics of DDTA in Figure 1a can be described by

(1) $$V_{w}=V_{y1}-V_{y2}+V_{y3}$$

(2) $$I_{o}=g_{m}\left(V_{w}-V_{y4}\right)$$

There are many applications of DDTA in the open literature, such as analog filters [13,14,15,16,17,18]. The universal filter in [13] uses DDTA with ±2 V supply and 1.66 mW power consumption. The analog wave filter in [14] uses DDTA with ±1.8 V supply and 21.59 μW power consumption. The mixed-mode universal filter in [15] uses DDTA with 1.2 V supply and 66 μW power consumption. The sub-volt universal filters in [16,17] use DDTAs with 0.5 V supply and 205.5 nW [16] and 277 nW [17] power consumption. Finally, the sub-voltage universal filter in [18] use DDTA with a 0.3 V supply and 357.4 nW power consumption.

Universal analog filters can be applied in telecommunication, electronic, and control systems, performing such functions as rejection of out-of-band noise, attenuation of the unwanted frequency components from the applied signal, realization of the active crossover network, or reduction of noise in a process measurement signal [19,20,21]. Moreover, they can be used to realize high-order filters [22]. In most applications of universal analog filters, voltage-mode analog filters with high-input and low-output impedances are required to avoid additional buffers or loading effects. There are universal analog filters with high-input and low-output impedances available in literature, using alternative active elements such as DDCCs [11,23], fully differential second-generation current conveyors (FDCCII) [24], current-feedback amplifiers (CFA) [25,26,27], universal voltage conveyors (UVC) [28], voltage differencing differential difference amplifier (VDDDA) [29]. However, analog filters based on these active devices in [11,23,24,25,26,27,28] require passive resistors and do not offer electronic tuning ability. The analog filters based on VDDDAs [29] provide an electronic tuning ability, but only voltage-mode filters and seven transfer functions are proposed.

This work proposes a versatile analog filter based on low-voltage differential difference transconductance amplifiers with enhanced performance. The proposed filter topology offers high-input and low-output impedances which is convenient for voltage-mode circuits, as well as many transfer functions of low-pass (LP), high-pass (HP), band-pass (BP), band-stop (BS) and all-pass (AP) filters into same topology. In addition, using the same versatile topology, transconductance-mode filters and a quadrature oscillator can be obtained. TM version provides LP, HP, BP, BS, and AP characteristics with high-input and high-output impedance, that is required of TM circuits. The natural frequency of the filters and the condition of oscillation for oscillator can be electronically controlled. The proposed DDTA and its applications operate with 0.5 V supply and are designed and simulated in the Cadence program using the 0.18 µm CMOS process from TSMC.

## 2. Proposed Circuit

### 2.1. 0.5 V DDTA

**Figure 2:**
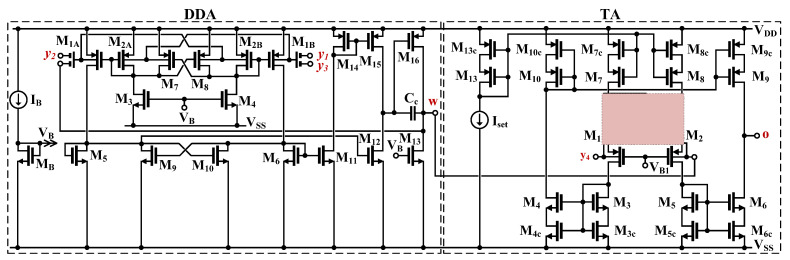
The CMOS structure of the 0.5 V DDTA.

**Figure 3:**
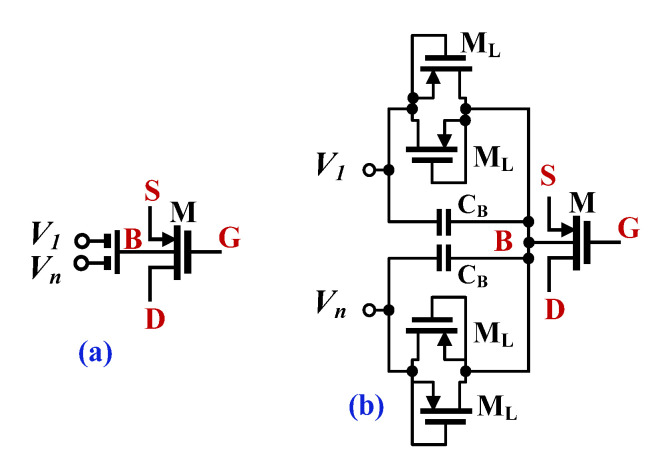
The symbol of bulk-driven MI-MOST (**a**) and the CMOS realization (**b**).

The CMOS structure of the proposed DDTA is shown in Figure 2. It consists of two main blocks, a differential-difference current conveyor realized with DDA operating in a negative-feedback configuration and TA [16]. The differential-difference amplifier is realized using a non-tailed differential pair M_1_-M_2_ [30], which is well suited for low-voltage circuits. However, the input transistors of this pair were replaced by multiple-input bulk-driven transistors (BD MI-MOST), shown in Figure 3. The multiple-input bulk-driven transistors are realized with an additional capacitive voltage divider, consisting of the capacitors C_B_, shunted by anti-parallel connections of the transistors M_L_. Note, that M_L_ operate with V_GS_ = 0, thus realizing a very large resistance R_LARGE_. Their purpose is to provide proper biasing of the input terminals for DC. For frequencies much larger than 1/R_LARGE_C_B_, the resulting impedance of the R_LERGE_C_B_ connections is dominated by capacitors, and the AC voltage at the bulk terminals of the multiple input transistors can be expressed as:

(3) $$V_{bi}=\sum_{i=1}^{n}\beta_{i}V_{i},$$

where, neglecting the impact of the MOS transistors, seen from their bulk terminals, the voltage gain of the input capacitive divider, from i-th input βi, in general case can be expressed as:

(4) $$\beta_{i}=\frac{C_{Bi}}{\sum_{i=1}^{n}C_{Bi}}$$

where n is the number of inputs. In the proposed design n = 2 and all capacitors C_B_ are equal to each other, then β_i_ = 1/2, for i = 1,2. Denoting the non-inverting and inverting input voltages of the input differential pair as V_i+_ and V_i-_, respectively, the differential voltage at the bulk terminals of the input non-tailed pair M_1A_ and M_1B_ can be expressed as:

(5) $$V_{bi}=\sum_{i=1}^{n}\beta_{i}\left(V_{+i}-V_{-i}\right),$$

Thus, the AC differential voltage of the “internal” differential pair is a sum of the input differential voltages applied to the capacitive inputs. In such a way, a differential-difference amplifier is realized, using only one transistor structure of the input pair, thus saving the dissipation power and decreasing complexity of the input stage.

Overall, the DDA used in the first block of the proposed DDTA can be seen as a two-stage amplifier, where the first stage can be considered as a current mirror OTA, based on the multiple-input pair, while the second stage, M_13_-M_16_, is a typical class-A common source amplifier. The capacitor C_C_ is used for frequency compensation. The two-stage architecture allows increasing the open-loop voltage gain and consequently the accuracy of the realized circuit function. The voltage gain is further increased thanks to the partial-positive feedback (PPF) circuit, realized using two sub-circuits M_7_-M_8_ and M_9_-M_10_. Each of the cross-coupled pairs of transistors generates negative resistances, thus partially increasing the resulting resistances at the gate/drain nodes of the transistors M_2A,B_ and M_5,6_ respectively, and consequently increasing the voltage gain of the DDA. However, PPF leads to increased sensitivity of the circuit to transistor mismatch, that can lead to stability problems. This sensitivity, like the voltage gain, increases with the amount of positive feedback, i.e., when the ratio of transconductances g_m7,8_/g_m2A,B_ (g_m9,10_/g_m5,6_) increases towards unity. Therefore, there is a tradeoff between the achieved improvement of the voltage gain and the circuit sensitivity to transistor mismatch. As it was shown in [16], applying two PPF circuits with weaker positive feedback, leads to the same improvement of the voltage gain, with less sensitivity, than applying one PPF circuit providing the same improvement of the gain. Therefore, in the circuit of Figure 2 two PPF circuits have been applied, one connected directly to the input transistors and the second, applied to the load of the input pair.

The open-loop low-frequency voltage gain of the DDA, from one differential input, with the second input grounded for AC signals, can be expressed as [16]:

(6) $$A_{vo}=\frac{\beta A_{o}}{\left(1-m_{1}\right)\left(1-m_{2}\right)}$$

where A_o_ is the DC open-loop voltage gain of the DDA without PPF circuits, calculated from the bulk terminals of M_1_, and given as:

(7) $$2g_{mb1}(r_{ds15}||r_{ds12})g_{m16}(r_{ds16}||r_{ds13})$$

The coefficients m_1_ and m_2_ can be expressed as:

(8) $$m_{1}=\frac{g_{m9,10}}{g_{m5,6}+g_{ds2}+g_{ds3,4}+g_{ds7,8}}≅\frac{g_{m9,10}}{g_{m5,6}}$$

(9) $$m_{2}=\frac{g_{m7,8}}{g_{m2}+g_{ds1}+g_{ds5,6}+g_{ds9,10}}≅\frac{g_{m7,8}}{g_{m2}}$$

The coefficients m_1_ and m_2_ can be considered as the ratios of negative to positive conductances in “bottom” and “upper” PPF. In general case, the coefficients can range from zero (lack of positive feedback) to unity (100% positive feedback). The overall voltage gain increases to infinity, as m_1_ or m_2_ tends to unity, i.e., as the amount of positive feedback increases. Thanks to the application of two PPFs in the proposed design with m_1_ = m_2_ = 0.5, a sufficient increase of the voltage gain (12 dB), with acceptable circuit sensitivity to transistor mismatch was achieved.

The second block of the DDTA in Figure 2 is a transconductance amplifier. In the proposed design, a BD source-degenerative (SD) differential pair, with triode region BD transistors M_11_ and M_12_ has been applied, which allows increasing the linear range of the TA by about 3 times, as compared with the conventional BD pair used in [16]. Even better linearity could be achieved using a non-tailed pair with a linear resistor [18,31], but such a solution is not very suitable for transconductors operating in nS range, since it would require very large resistors, not practical in silicon realizations.

For the TA in Figure 2, operating in a weak-inversion region, optimum linearity is achieved if the following condition is satisfied:

(10) $$k=\frac{{\left(W/L\right)}_{11.12}}{{\left(W/L\right)}_{1,2}}=0.5$$

where W and L are the transistor channel width and length, respectively. Note, that the above condition is the same as for the gate-driven counterpart of the circuit [32]. For this optimum case, with unity-gain current mirrors, the circuit transconductance g_m_ can be expressed as:

(11) $$g_{m}=\eta \cdot \frac{4k}{4k+1}\cdot \frac{I_{set}}{n_{p}U_{T}}$$

where η = g_mb1,2_/g_m1,2_ is the bulk to gate transconductance ratio at the operating point for the input transistors M_1_ and M_2_, n_p_ is the subthreshold slope factor for p-channel MOS transistors and U_T_ is the thermal potential. As it can be concluded from (11), the circuit transconductance is proportional to the biasing current I_set_, thus it can be easily regulated using this current.

### 2.2. Versatile Analog Filter

**Figure 4:**
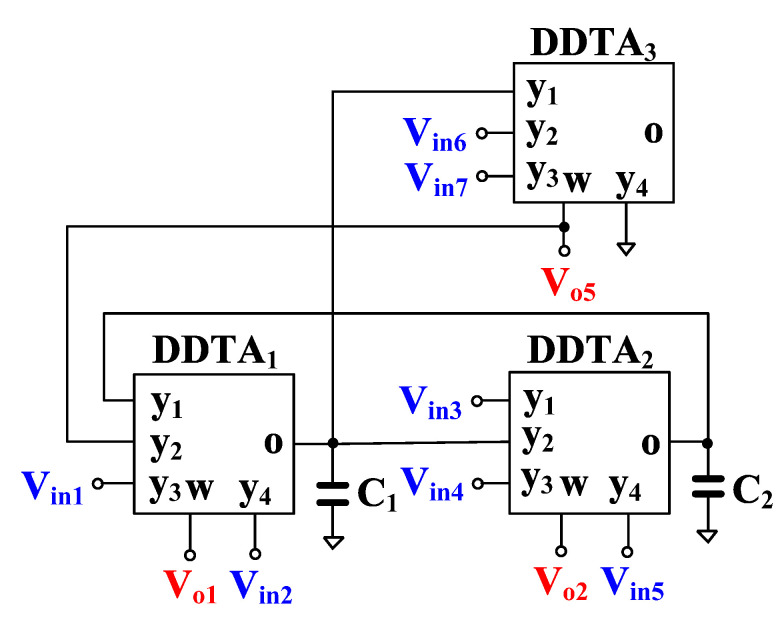
Proposed voltage-mode analog filter.

The proposed versatile analog filter is shown in Figure 4. The circuit employs only three DDTAs and two grounded capacitors. It should be noted that the output voltages V_o1_, V_o2_, and V_o3_ are defined at the low-impedance w-terminals while the input voltages V_in1_ to V_in7_ are fed to the high-impedance y-terminals of the DDTA.

Using (1), (2) and nodal analysis, the output voltages of the circuit in Figure 4 can be expressed by

(12) $$V_{o1}=\frac{s^{2}C_{1}C_{2}\left(V_{in1}+V_{in6}-V_{in7}\right)+\left(sC_{2}g_{m1}+g_{m1}g_{m2}\right)V_{in2}+sC_{1}g_{m2}\left(V_{in3}+V_{in4}-V_{in5}\right)}{S^{2}C_{1}C_{2}+SC_{2}g_{m1}+g_{m1}g_{m2}},$$

(13) $$V_{o2}=\frac{sC_{2}g_{m1}\left(-V_{in1}+V_{in2}-V_{in6}+V_{in7}\right)+\left(S^{2}C_{1}C_{2}+SC_{2}g_{m1}\right)\left(V_{in3+}V_{in4}\right)+g_{m1}g_{m2}V_{in5}}{S^{2}C_{1}C_{2}+SC_{2}g_{m1}+g_{m1}g_{m2}},$$

(14) $$V_{o3}=\frac{sC_{2}g_{m1}\left(V_{in1}-V_{in2}\right)+g_{m1}g_{m2}\left(V_{in3}+V_{in4}-V_{in5}\right)+\left(s^{2}C_{1}C_{2}+g_{m1}g_{m2}\right)(V_{in7}-V_{in6})}{S^{2}C_{1}C_{2}+SC_{2}g_{m1}+g_{m1}g_{m2}},$$

The filtering responses can be obtained by appropriately applying the input signals and choosing the output voltages and the variant filtering responses are shown in Table 1. It should be noted that the VM analog filter provides 34 filtering responses of LP, HP, BP, BS, and AP filters, all of them in both, inverting and non-inverting versions.

Considering denominator of (12)–(14), the natural frequency (*ω_o_*) and the quality factor (*Q*) are given by

(15) $$\omega_{o}=\sqrt{\frac{g_{m1}g_{m2}}{C_{1}C_{2}}},$$

(16) $$Q=\sqrt{\frac{g_{m2}C_{1}}{g_{m1}C_{2}}}$$

The parameter ω_o_ can be controlled electronically by g_m1_ = g_m2_ and, in this case, the parameter *Q* is given by C_1_/C_2_.

**Figure 5:**
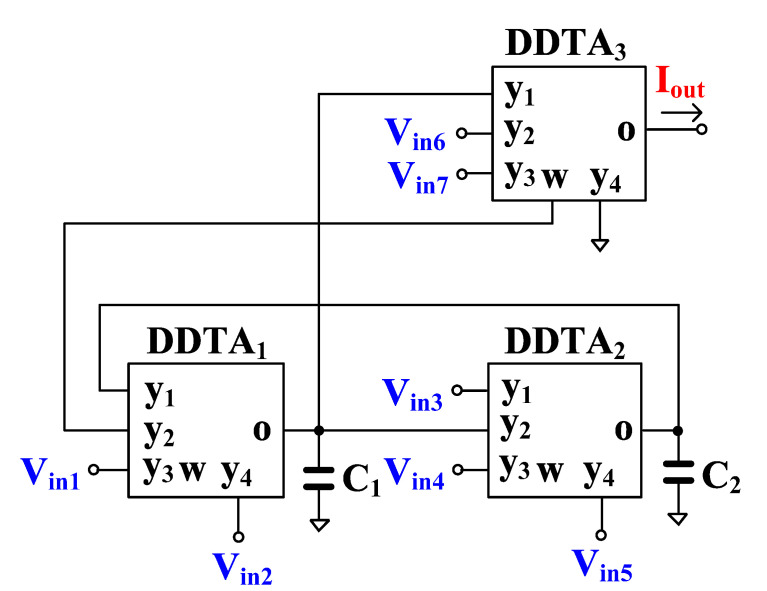
Modified transconductance-mode analog filter.

The VM analog filter in Figure 4 can also operate as transconductance-mode (TM) analog filter, as shown in Figure 5. The output current of the TM filter is the output current of the o-terminal of DDTA_3_ while the input voltages are same as for the VM filter. The output current of the TM filter is given by

(17) $$I_{out}=\frac{\begin{array}{c} sC_{2}g_{m1}g_{m3}\left(V_{in1}-V_{in2}\right)+g_{m1}g_{m2}g_{m3}\left(V_{in3}+V_{in4}-V_{in5}\right)\\ +\left(s^{2}C_{1}C_{2}+g_{m1}g_{m2}\right)g_{m3}\left(V_{in7}-V_{in6}\right) \end{array}}{s^{2}C_{1}C_{2}+sC_{2}g_{m1}+g_{m1}g_{m2}}$$

The filtering responses can be obtained by appropriately applying the input signals. The variant filtering responses are listed in Table 2. It should be noted that the TM filter provides 11 transfer functions of LP, HP, BP, BS, and AP filters and all filtering responses provide both inverting and non-inverting transfer functions.

**Figure 6:**
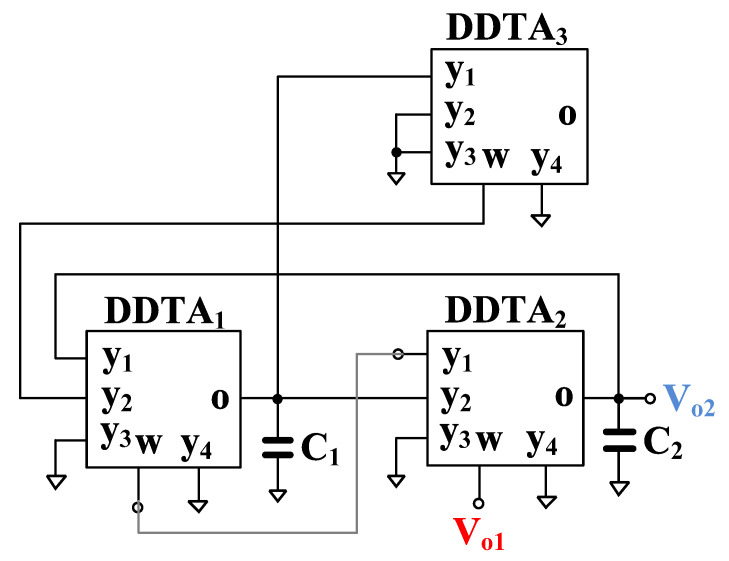
Modified quadrature oscillator.

The proposed VM analog filter can be modified to work as a quadrature oscillator as shown in Figure 6. The VM transfer function of a BP filter V_o1_/V_in3_ is used, with a gain given by

(18) $$\frac{V_{o1}}{V_{in3}}=\frac{sC_{1}g_{m2}}{s^{2}C_{1}C_{2}+sC_{2}g_{m1}+g_{m1}g_{m2}}$$

Assuming V_o1_/V_in3_ = 1, the characteristic equation of the oscillator is

(19) $$s^{2}C_{1}C_{2}+s\left(C_{2}g_{m1}-C_{1}g_{m2}\right)+g_{m1}g_{m2}=0$$

assuming further g_m1_ = g_m2_, the condition of oscillation is given by

(20) $$C_{2}-C_{1}=0$$

The frequency of oscillation is expressed as

(21) $$\omega_{o}=\sqrt{\frac{g_{m1}g_{m2}}{C_{1}C_{2}}}$$

Note, that the frequency of oscillation *ω_o_* can be electronically controlled via g_m1_ and g_m2_.

Considering nodes V_o1_ and V_o2_ in Figure 6, we see, that DDTA_2_ and C_2_ form a lossless integrator with a transfer function given by

(22) $$\frac{V_{o2}}{V_{o1}}=\frac{g_{m2}}{sC_{2}}$$

At *ω* = *ω_o_*, the phase and magnitude are given respectively by ∅ = π/2 and |g_m2_/C_2_|.

**Non-idealities analysis**

In non-ideal case, the main equations describing the characteristics of DDTA can be expressed as:

(23) $$V_{w}=\beta_{k1}V_{y1}-\beta_{k2}V_{y2}+\beta_{k3}V_{y3}$$

(24) $$I_{o}=g_{mnk}\left(V_{w}-V_{y4}\right)$$

where *β_k1_*, *β_k2_*, *β_k3_* denote the non-ideal voltage gains from y_1_, y_2_ and y_3_ terminals, respectively, to the w-terminal of the k-th DDTA (*β_k1_* = *β_k2_* = *β_k3_* = 1 in ideal case), g_mnk_ is the frequency-dependent transconductance of the k-th DDTA. At the frequency near the cut-off frequency, g_mnk_ can be approximated by [33]

(25) $$g_{mnk}=g_{mk}\left(1-\mu_{k}s\right),$$

where μ_k_ = 1⁄ω_gk_, and ω_gk_ denotes the first pole of the k-th DDTA.

Using (23), the output voltages V_o1_, V_o2_, and V_o3_ of the VM analog filter can be rewritten as

(26) $$V_{o1}=\frac{\begin{array}{c} \left\{s^{2}C_{1}C_{2}\left(\beta_{32}V_{in1}+\beta_{12}\beta_{33}V_{in6}-\beta_{12}\beta_{31}V_{in7}\right)+\left(sC_{2}g_{mn1}\beta_{12}\beta_{31}+g_{mn1}g_{mn2}\beta_{11}\beta_{22}\right)V_{in2}\right.\\ \left.+sC_{1}g_{mn2}\left(\beta_{11}\beta_{21}V_{in3}+\beta_{11}\beta_{23}V_{in4}-\beta_{12}V_{in5}\right)\right\} \end{array}}{s^{2}C_{1}C_{2}+sC_{2}g_{mn1}\beta_{12}\beta_{31}+g_{mn1}g_{mn2}\beta_{11}\beta_{22}},$$

(27) $$V_{o2}=\frac{\begin{array}{c} \left\{sC_{2}g_{mn1}(-\beta_{13}\beta_{22}V_{in1}+\beta_{22}V_{in2}-\beta_{12}\beta_{22}\beta_{32}V_{in6}+\beta_{12}\beta_{22}\beta_{33}V_{in7})\right.\\ \left.+\left(s^{2}C_{1}C_{2}+sC_{2}g_{mn1}\beta_{12}\beta_{31}\right)\left(\beta_{21}V_{in3+}\beta_{23}V_{in4}\right)+g_{mn1}g_{mn2}\beta_{11}\beta_{22}V_{in5}\right\} \end{array}}{s^{2}C_{1}C_{2}+sC_{2}g_{mn1}\beta_{12}\beta_{31}+g_{mn1}g_{mn2}\beta_{11}\beta_{22}},$$

(28) $$V_{o3}=\frac{\begin{array}{c} sC_{2}g_{mn1}\left\{\left(\beta_{13}\beta_{31}V_{in1}-\beta_{31}V_{in2}\right)+g_{mn1}g_{mn2}\left(\beta_{11}\beta_{21}\beta_{31}V_{in3}+\beta_{11}\beta_{23}\beta_{31}V_{in4}-\beta_{11}\beta_{31}V_{in5}\right)\right.\\ \left.+\left(s^{2}C_{1}C_{2}+g_{mn1}g_{mn2}\beta_{11}\beta_{22}\right)\left(\beta_{32}V_{in7}-\beta_{33}V_{in6}\right)\right\} \end{array}}{s^{2}C_{1}C_{2}+sC_{2}g_{mn1}\beta_{12}\beta_{31}+g_{mn1}g_{mn2}\beta_{11}\beta_{22}},$$

Furthermore, the output current I_out_ of the TM analog filter can be rewritten as

(29) $$I_{out}=\frac{\begin{array}{c} \left\{sC_{2}g_{mn1}g_{mn3}\left(\beta_{13}\beta_{31}V_{in1}-\beta_{31}V_{in2}\right)+g_{mn1}g_{mn2}g_{mn3}\left(\beta_{11}\beta_{21}\beta_{31}V_{in3}+\beta_{11}\beta_{23}\beta_{31}V_{in4}-\beta_{11}\beta_{31}V_{in5}\right)\right.\\ \left.+\left(s^{2}C_{1}C_{2}+g_{mn1}g_{mn2}\beta_{11}\beta_{22}\right)g_{mn3}\left(\beta_{32}V_{in7}-\beta_{33}V_{in6}\right)\right\} \end{array}}{s^{2}C_{1}C_{2}+sC_{2}g_{mn1}\beta_{12}\beta_{31}+g_{mn1}g_{mn2}\beta_{11}\beta_{22}},$$

Using (25), the denominators of (26)–(29) can be expressed by

(30) $$\begin{array}{c} s^{2}C_{1}C_{2}\left(1-\frac{C_{2}g_{m1}\beta_{12}\beta_{31}\mu_{1}-\beta_{11}\beta_{22}\mu_{1}\mu_{2}}{C_{1}C_{2}}\right)\\ \;\;\;\;\;\;\;\;\;\;\;\;+sC_{2}g_{m1}\beta_{12}\beta_{31}\left(1-\frac{g_{m1}g_{m2}\beta_{11}\beta_{22}\mu_{1}+g_{m1}g_{m2}\beta_{11}\beta_{22}\mu_{2}}{C_{2}g_{m1}\beta_{12}\beta_{31}}\right)\\ \;\;\;\;\;\;\;\;\;\;\;\;+g_{m1}g_{m2}\beta_{11}\beta_{22} \end{array}$$

From (30), the parasitic effects from DDTA can be ignored if the following conditions are met

(31) $$\frac{C_{2}g_{m1}\beta_{12}\beta_{31}\mu_{1}-\beta_{11}\beta_{22}\mu_{1}\mu_{2}}{C_{1}C_{2}}\ll 1,$$

(32) $$\frac{g_{m1}g_{m2}\beta_{11}\beta_{22}\mu_{1}+g_{m1}g_{m2}\beta_{11}\beta_{22}\mu_{2}}{C_{2}g_{m1}\beta_{12}\beta_{31}}\ll 1$$

The non-ideal parameters *ω_o_* and *Q* can be expressed as

(33) $$\omega_{o}=\sqrt{\frac{g_{m1}g_{m2}\beta_{11}\beta_{22}}{C_{1}C_{2}}},$$

(34) $$Q=\sqrt{\frac{g_{m2}C_{1}\beta_{11}\beta_{22}}{g_{m1}C_{2}\beta_{12}\beta_{31}}}$$

From the BP function (26), with V_o1_/V_in3_ = 1, the non-ideal characteristic equation of the oscillator can be expressed by

(35) $$s^{2}C_{1}C_{2}+s\left(C_{2}g_{mn1}\beta_{12}\beta_{31}-C_{1}g_{mn2}\beta_{11}\beta_{21}\right)+g_{mn1}g_{mn2}\beta_{11}\beta_{22}=0$$

The condition of oscillations and the frequency of oscillations are:

(36) $$C_{2}g_{mn1}\beta_{12}\beta_{31}-C_{1}g_{mn2}\beta_{11}\beta_{21}=0,$$

(37) $$\omega_{o}=\sqrt{\frac{g_{mn1}g_{mn2}\beta_{11}\beta_{22}}{C_{1}C_{2}}}$$

## 3. Simulation Results

The circuit was designed and simulated in the Cadence environment using the 0.18 µm CMOS technology form TSMC. The transistors aspect ratios are in Table 3. The voltage supply V_DD_ = 0.5 V and the bias voltage V_B1_ = −60 mV. For I_set_ = 5 nA, the total power consumption of the DDTA was 215.5 nW (DDA = 203 nW and TA = 12.5 nW). It is worth mentioning that the DC level on the bulk terminal of the differential pair depends on the DC level of the input signals and on the shunt resistors M_L_ that form a resistor voltage divider [34]. The simulated current of the bulk terminal of the differential pair is less than 0.8% of the input currents in the wholly input range, and hence the current of the bulk terminal could be neglected compared to the inputs one [34]. The value of this input capacitor C_B_ was optimized, based on previous post-layout simulation, to be 0.5 pF, in order to reduce the impact of the parasitic capacitance of the MOS transistor on the circuit performance from one side and to avoid extra increase in chip area from the other side [34,35]. The performance of the MI-MOST was confirmed experimentally in [34,35].

**Figure 7:**
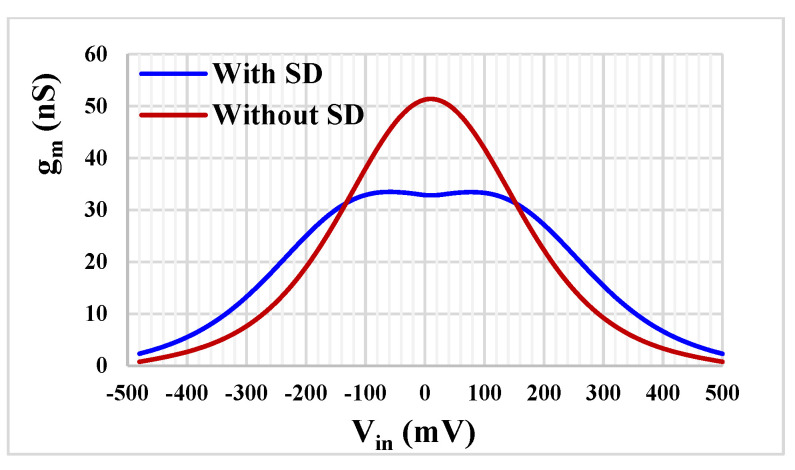
The transconductance characteristics of the TA with and without SD technique.

Figure 7 shows the transconductance characteristics versus input voltage of the TA with the proposed SD and without SD that was presented in [16] for I_set_ = 5 nA. While the transconductance varies by about 10% over the nominal value for the input voltage range of ±40 mV for TA without SD, it is up to ±160 mV for the proposed TA with the SD. Note the improved linearity, obtained thanks to the SD technique.

**Figure 8:**
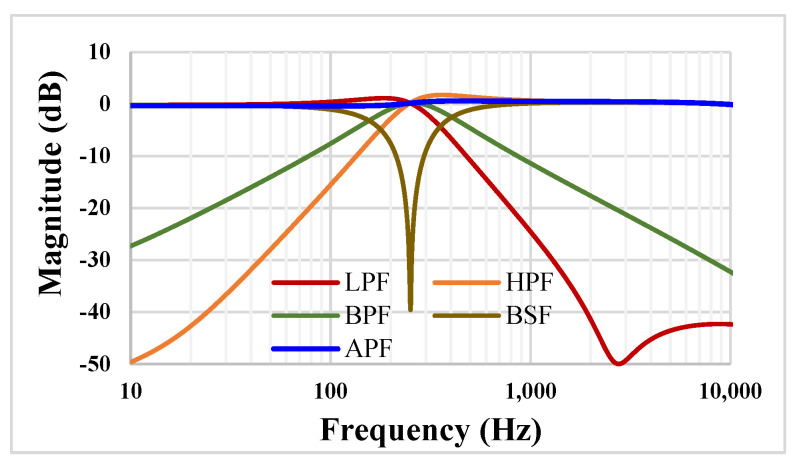
The frequency characteristics of the VM filter.

The frequency characteristics for the proposed VM filter from Figure 4 are shown in Figure 8. The value of the capacitors were C_1_ = C_2_ = 20 pF and the setting current I_set_ = 5 nA. The −3 dB cut-off frequency for the LP filter was 323.3 Hz.

**Figure 9:**
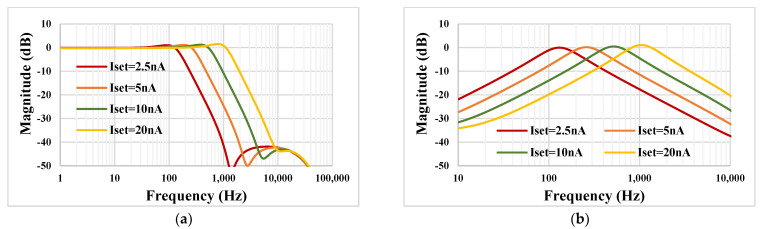
The simulated frequency characteristics showing the tuning capability of the LP (**a**) and BP filters (**b**).

The tuning capability for the selected LP and BP filters are shown in Figure 9. With I_set_ = 2.5 nA, 5 nA, 10 nA, 20 nA the −3 dB frequency of the LPF was 162 Hz, 323.3 Hz, 650.2 Hz and 1.333 kHz, respectively. This, for example, covers a wide spectrum of biosignal filtering applications.

**Figure 10:**
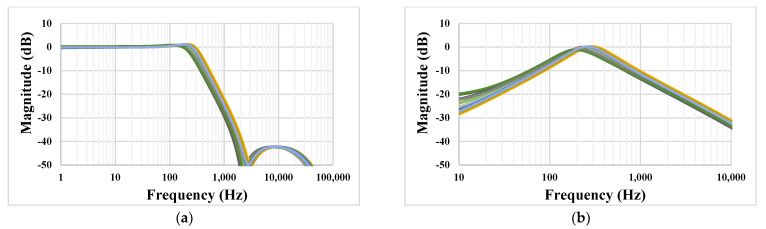
The simulated frequency characteristic of the LP (**a**) and BP (**b**) filters with PVT corners.

The simulated frequency characteristic of the LP (a) and BP (b) filters with process, voltage, and temperature (PVT) corners are shown in Figure 10. The process corners were fast–fast, fast–slow, slow–fast and slow–slow, the temperature corners were −10 °C and 70 °C, and the voltage supply corners were ±10%V_DD_. The variation of these characteristics is in acceptable range.

**Figure 11:**
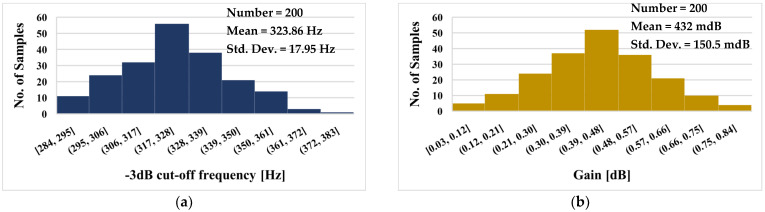
The histogram of the −3 dB cut-off frequency (**a**) and low frequency gain (**b**) of the LPF with 200 MC runs.

The histograms of the −3 dB cut-off frequency and low frequency gain of the LP filter with Monte Carlo (MC) process and mismatch analysis are shown in Figure 11a and Figure 11b, respectively. For the −3 dB cut-off frequency, the mean value was 323.86 Hz and the standard deviation 17.95 Hz, while the mean value of the gain was 432 mdB with a standard deviation of 150.5 mdB. Note that, thanks to the electronic tuning ability of the DDTA, any possible deviation of the filter parameters could be readjusted by the setting current I_set_.

**Figure 12:**
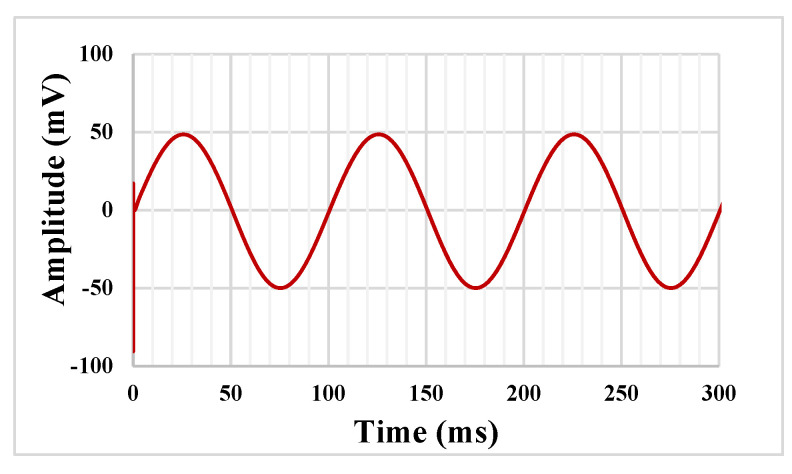
The transient response of the LP filter.

**Figure 13:**
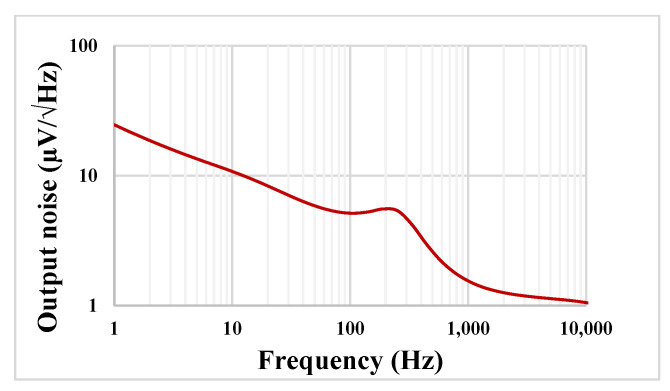
The output noise the LP filter.

The transient response of the LP filter with applied input sine wave signal 100 mV_pp_ @ 10 Hz is shown in Figure 12. The total harmonic distortion (THD) was 0.8%. The output noise of the LPF is shown in Figure 13. The integrated noise value was 108 µV, that results in a dynamic range of 53.2 dB for 1% THD.

**Figure 14:**
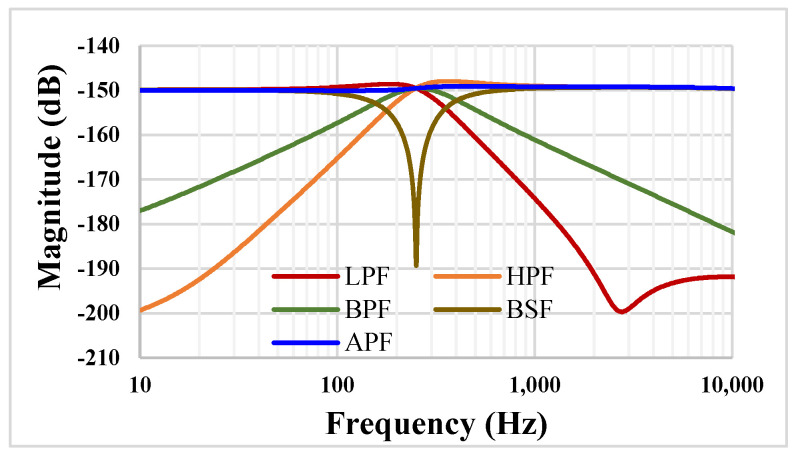
The frequency characteristics of the TM filter.

The frequency characteristics for the TM filter from Figure 5 are shown in Figure 14 for same condition as for the VM filter i.e., C_1_ = C_2_ = 20 pF and I_set_ = 5 nA. The low frequency current gain of the LP filter was −150 dB, which corresponds to a transconductance 31.6 nS, and the −3 dB for the LPF was 323.3 Hz.

**Figure 15:**
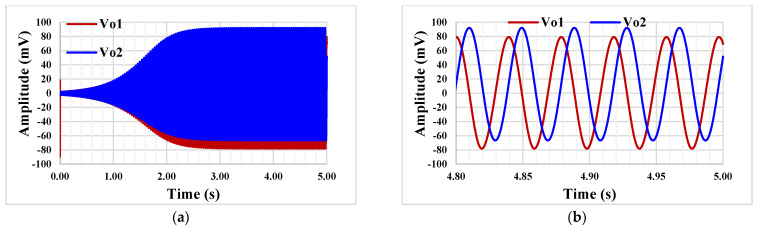
The starting oscillation (**a**) and the steady state (**b**).

The quadrature oscillator in Figure 6 was also simulated with I_set_ = 5 nA and capacitor values C_1_ = C_2_ = 20 pF. Figure 15 shows the starting oscillation (a) and the steady state (b), respectively. The frequency is 253 Hz and the THD was around 1%.

Finally, Table 4 provides the performance comparison of this work with others recently published works [11,15,16,18]. This universal filter offers high-input and low-output impedances of VM filter and high-input and high-output impedances of TM filter. Compared with [11,15,16,18], the proposed filters offer 34 transfer functions of VM filter and 11 transfer functions of TIM filters. Compared with [11], the proposed filter provides electronic tuning ability of natural frequency and low-power consumption. Compared with the DDTA-based analog filters in [15,16,17,18], the proposed filter offers the advantages such as low-output impedances which is required for VM circuits, both non-inverting and inverting transfer functions of LP, HP, BP, BS, and AP filters, and maximum VM transfer functions.

## 4. Conclusions

This paper presents a voltage-, transconductance-mode analog filter and quadrature oscillator based on low-voltage low-power DDTA. These applications require three DDTAs and two grounded capacitors, which is suitable for integrated circuit implementation. Both VM and TM filters provide five standard filtering responses, namely, low-pass, high-pass, band-pass, band-stop and all-pass responses into single topology. The natural frequency of these filter responses and the condition of oscillation can be electronically controlled. The provided simulation including Monte Carlo and PVT corners confirm the advantages and stability of the proposed applications.

## Figures and Tables

**Table 1:** Obtaining variant filtering function of voltage-mode analog filter.

Output	Input	Filtering Function	Transfer Functions
$V_{o1}$	$V_{in2}=V_{in5}=V_{in}$	Non-inverting LP	$g_{m1}g_{m2}/D\left(s\right)$
	$V_{in3}=V_{in}$	Non-inverting BP	$sC_{1}g_{m2}/D\left(s\right)$
	$V_{in4}=V_{in}$	Non-inverting BP	$sC_{1}g_{m2}/D\left(s\right)$
	$V_{in5}=V_{in}$	Inverting BP	$-sC_{1}g_{m2}/D\left(s\right)$
	$V_{in1}=V_{in}$	Non-inverting HP	$s^{2}C_{1}C_{2}/D\left(s\right)$
	$V_{in6}=V_{in}$	Non-inverting HP	$s^{2}C_{1}C_{2}/D\left(s\right)$
	$V_{in7}=V_{in}$	Inverting HP	$-s^{2}C_{1}C_{2}/D\left(s\right)$
	$V_{in1}=V_{in2}=V_{in5}=V_{in}$	Non-inverting BS	$s^{2}C_{1}C_{2}+g_{m1}g_{m2}$
	$V_{in2}=V_{in5}=V_{in6}=V_{in}$	Non-inverting BS	$s^{2}C_{1}C_{2}+g_{m1}g_{m2}$
$V_{o2}$	$V_{in5}=V_{in}$	Non-Inverting LP	$g_{m1}g_{m2}/D\left(s\right)$
	$V_{in1}=V_{in}$	Inverting BP	$-sC_{2}g_{m1}/D\left(s\right)$
	$V_{in2}=V_{in}$	Non-inverting BP	$sC_{2}g_{m1}/D\left(s\right)$
	$V_{in6}=V_{in}$	Inverting BP	$-sC_{2}g_{m1}/D\left(s\right)$
	$V_{in7}=V_{in}$	Non-inverting BP	$sC_{2}g_{m1}/D\left(s\right)$
	$V_{in1}=V_{in3}=V_{in}$	Non-inverting HP	$s^{2}C_{1}C_{2}/D\left(s\right)$
	$V_{in1}=V_{in4}=V_{in}$	Non-inverting HP	$s^{2}C_{1}C_{2}/D\left(s\right)$
	$V_{in3}=V_{in6}=V_{in}$	Non-inverting HP	$s^{2}C_{1}C_{2}/D\left(s\right)$
	$V_{in4}=V_{in6}=V_{in}$	Non-inverting HP	$s^{2}C_{1}C_{2}/D\left(s\right)$
	$V_{in1}=V_{in3}=V_{in5}=V_{in}$	Non-inverting BS	$s^{2}C_{1}C_{2}+g_{m1}g_{m2}/D\left(s\right)$
	$V_{in1}=V_{in4}=V_{in5}=V_{in}$	Non-inverting BS	$s^{2}C_{1}C_{2}+g_{m1}g_{m2}/D\left(s\right)$
	$V_{in3}=V_{in5}=V_{in6}=V_{in}$	Non-inverting BS	$s^{2}C_{1}C_{2}+g_{m1}g_{m2}/D\left(s\right)$
	$V_{in4}=V_{in5}=V_{in6}=V_{in}$	Non-inverting BS	$s^{2}C_{1}C_{2}+g_{m1}g_{m2}/D\left(s\right)$
	$V_{in3}=V_{in5}=V_{in1}=V_{in6}=V_{in}$	Non-inverting AP	$s^{2}C_{1}C_{2}-sC_{2}g_{m1}+g_{m1}g_{m2}/D\left(s\right)$
$V_{o3}$	$V_{in3}=V_{in}$	Non-inverting LP	$g_{m1}g_{m2}/D\left(s\right)$
	$V_{in4}=V_{in}$	Non-inverting LP	$g_{m1}g_{m2}/D\left(s\right)$
	$V_{in5}=V_{in}$	Inverting LP	$-g_{m1}g_{m2}/D\left(s\right)$
	$V_{in1}=V_{in}$	Non-inverting BP	$sC_{2}g_{m1}/D\left(s\right)$
	$V_{in2}=V_{in}$	Inverting BP	$-sC_{2}g_{m1}/D\left(s\right)$
	$V_{in7}=V_{in5}=V_{in}$	Non-inverting HP	$s^{2}C_{1}C_{2}/D\left(s\right)$
	$V_{in3}=V_{in6}=V_{in}$	Inverting HP	-$s^{2}C_{1}C_{2}/D\left(s\right)$
	$V_{in7}=V_{in}$	Non-inverting BS	$s^{2}C_{1}C_{2}+g_{m1}g_{m2}/D\left(s\right)$
	$V_{in6}=V_{in}$	Inverting BS	$-\left(s^{2}C_{1}C_{2}+g_{m1}g_{m2}\right)/D\left(s\right)$
	$V_{in2}=V_{in7}=V_{in}$	Non-inverting AP	$s^{2}C_{1}C_{2}-sC_{2}g_{m1}+g_{m1}g_{m2}/D\left(s\right)$
	$V_{in1}=V_{in6}=V_{in}$	Inverting AP	$-\left(s^{2}C_{1}C_{2}-sC_{2}g_{m1}+g_{m1}g_{m2}\right)/D\left(s\right)$

where $D\left(s\right)=s^{2}C_{1}C_{2}+sC_{2}g_{m1}+g_{m1}g_{m2}$.

**Table 2:** Obtaining variant filtering function of transconductance-mode analog filter.

Output	Input	Filtering Function	Transfer Functions
$I_{out}$	$V_{in3}=V_{in}$	Non-inverting LP	$g_{m1}g_{m2}g_{m3}/D\left(s\right)$
	$V_{in4}=V_{in}$	Non-inverting LP	$g_{m1}g_{m2}g_{m3}/D\left(s\right)$
	$V_{in5}=V_{in}$	Inverting LP	$-g_{m1}g_{m2}g_{m3}/D\left(s\right)$
	$V_{in1}=V_{in}$	Non-inverting BP	$sC_{2}g_{m1}g_{m3}/D\left(s\right)$
	$V_{in2}=V_{in}$	Inverting BP	$-sC_{2}g_{m1}g_{m3}/D\left(s\right)$
	$V_{in7}=V_{in5}=V_{in}$	Non-inverting HP	$s^{2}C_{1}C_{2}/D\left(s\right)$
	$V_{in3}=V_{in6}=V_{in}$	Inverting HP	$s^{2}C_{1}C_{2}/D\left(s\right)$
	$V_{in6}=V_{in}$	Inverting BS	$\{(s{\}}^{2}C_{1}C_{2}+g_{m1}g_{m2})/D\left(s\right)$
	$V_{in7}=V_{in}$	Non-inverting BS	$-\{(s{\}}^{2}C_{1}C_{2}+g_{m1}g_{m2})g_{m3}/D\left(s\right)$
	$V_{in2}=V_{in7}=V_{in}$	Non-inverting AP	$\{(s{\}}^{2}C_{1}C_{2}-sC_{2}g_{m1}+g_{m1}g_{m2})g_{m3}/D\left(s\right)$
	$V_{in1}=V_{in6}=V_{in}$	Inverting AP	$-\{(s{\}}^{2}C_{1}C_{2}+sC_{2}g_{m1}+g_{m1}g_{m2})g_{m3}/D\left(s\right)$

where $D\left(s\right)=s^{2}C_{1}C_{2}+sC_{2}g_{m1}+g_{m1}g_{m2}$.

**Table 3:** Transistor aspect ratio of the DDTA.

DDA	W/L (µm/µm)	TA	W/L (µm/µm)
M_1A_, M_2A_, M_1B_, M_2B_ M_14_, M_15_	16/3	M_1_, M_2_	5 × 15/1
M_3_-M_8_, M_11_-M_12_, M_B_	8/3	M_3_-M_6_	2 × 10/1
M_9_, M_10_	4/3	M_3c_-M_6c_	10/1
M_16_	6 × 16/3	M_8_, M_9_, M_B1_, M_11_, M_12_	2 × 15/1
M_13_	6 × 8/3	M_8c_, M_9c_, M_B1c_	15/1
M_L_	4/5	M_7_	2 × 30/1
MIM capacitor: C_B_ = 0.5 pF, C_c_ = 6 pF		M_7c_	30/1

**Table 4:** Performance comparison of this work with those of recently published.

Factor	[11]	[15]	[16]	[18]	Proposed
Number of active devices	3 DDCC	5-DDTA	3 DDTA	2 DDTA	3 DDTA
Realization	130 nm	180 nm	130 nm	130 nm	180 nm
Number of passive devices	2 R, 2 C	2 C	2 C	2 C	2 C
Type of filter	MISO	MIMO	MIMO	MIMO	MIMO
Operation mode	VM	VM/CM/TAM/TIM	VM	VM	VM/TIM
Number of offered responses	5 (VM)	36 (VM/CM/TAM/TIM)	23 (VM)	22 (VM)	34 (VM) 11 (TIM)
Active device offers electronic control	No	Yes	Yes	Yes	Yes
High-input and low-output impedance of VM	Yes	No	No	No	Yes
High-input and high-output impedance of TM	-	Yes	-	-	Yes
Orthogonal control of $\omega_{o}$ and Q	Yes	Yes	Yes	Yes	Yes
Electronic control of $\omega_{o}$	No	Yes	Yes	Yes	Yes
Offer modified into oscillator	No	No	No	Yes	Yes
Orthogonal control of CO and FO	-	-	-	Yes	Yes
Natural frequency (kHz)	6370	1.04	0.254	0.08147	0.323
Simulated power supply (V)	±0.75	1.2	0.5	0.3	0.5
Power dissipation (μW)	3650	330	0.616	0.715	0.646
THD (%)	3 @120 m V_pp_	1.09@650 mV_pp_	0.62 @100 m V_pp_	0.5 @100 m V_pp_	0.8@100 mV_pp_
Dynamic range (dB)	-	-	49.7	-	53.2
Verification of result	Sim/Exp	Sim/Exp	Sim	Sim	Sim
